# Combined study on clastogenic, aneugenic and apoptotic properties of doxorubicin in human cells in vitro

**DOI:** 10.1186/s40709-018-0089-z

**Published:** 2018-10-11

**Authors:** Vasiliki Chondrou, Katerina Trochoutsou, Andreas Panayides, Maria Efthimiou, Georgia Stephanou, Nikos A. Demopoulos

**Affiliations:** Division of Genetics, Cell and Developmental Biology, Department of Biology, University of Patras, 26 504, Patras, Greece

**Keywords:** Apoptosis-caspase-3, Doxorubicin, Fpg and hOGG1 comet assay, Micronucleation, Mitotic spindle disturbance

## Abstract

**Background:**

Doxorubicin is a widely used anticancer drug due to its broad spectrum of antitumor activity. Various mechanisms have been proposed for its cytostatic activity, including DNA intercalation, topoisomerase II inhibition, generation of free radicals and apoptosis. The present study aims to further clarify the cytostatic activity of doxorubicin by its specific effect on (a) DNA damage, (b) micronucleation and (c) apoptosis, using a combination of different methods and cell systems such as human lymphocytes and HL-60 human leukemic cells. DNA lesions were analyzed by the alkaline comet assay in combination with formamidopyrimidine (Fpg) and human 8-oxoguanine (hOGG1) repair enzymes. Micronucleation was investigated by the Cytokinesis-Block Micronucleus assay (CBMN) in combination with Fluorescence In Situ Hybridization analysis. Impairment on mitotic apparatus was investigated by double immunofluorescence of β- and γ-tubulin. Apoptotic cell frequency was determined by the CBMN cytome assay. Complementary to the above, caspase-3 level was investigated by Western blot.

**Results:**

It was found that doxorubicin generates DNA breakage induced by oxidative damage in DNA bases, which can be repaired by the Fpg and hOGG1 enzymes. Increased micronucleus frequency was identified mainly through chromosome breakage and, at a lesser extent, through chromosome delay. Analysis of mitotic spindle showed disturbance of chromosome orientation and centrosome duplication and/or separation, leading to aneuploidy. Enhanced frequency of apoptotic leukemic cells was also observed. Caspase-3 seems to be involved in the generation of apoptosis.

**Conclusions:**

The aforementioned findings derived from different treatment schedules, doses and time of exposure on primary versus transformed cells extend our knowledge about doxorubicin genotoxicity and contribute to the better understanding of the mechanisms by which doxorubicin induces genotoxic effects on human cells.

## Background

Doxorubicin (DOX) is a widely used anticancer drug due to its broad spectrum of antitumor activity. It belongs to the family of anthracyclines. Anthracyclines remain some of the most effective anticancer drugs and are crucial to the treatment of acute leukemia, Hodgkin’s and non-Hodgkin’s lymphoma, and breast cancer [1]. Various mechanisms have been proposed for DOX cytostatic activity, including DNA intercalation, topoisomerase II inhibition, generation of free radicals and apoptosis [2].

The comet assay or Single Cell Gel Electrophoresis (SCGE) assay is a rapid, sensitive, relatively simple and fairly inexpensive method for detecting DNA damage in individual cells. It is based on quantification of the denatured DNA fragments migrating as comet tail out of the cell nucleus during electrophoresis. This assay is used in human studies, ecological monitoring, genotoxicity testing and estimation of DNA repair [3–5]. The standard alkaline SCGE method gives limited information on the type of DNA damage being measured. It is not possible to determine whether this is a consequence of direct effects of the damaging agent, or of indirect effects such as oxidative damage, AP sites or DNA repair. The sensitivity and specificity of the assay can be improved by incubating the lysed cells (nucleoids) with lesion specific endonucleases, which recognize particular damaged bases and convert lesions to additional DNA breaks, increasing the amount of DNA in the comet tail [6].

Micronucleus test is one of the main methods in use to detect chromosome damage in human cells. Micronuclei (MN) are generated during cell division and consist of genetic material located inside the cytoplasm but outside of the nucleus. They may contain either acentric chromosome fragments or whole chromosomes, which are not incorporated to any of the daughter nuclei during anaphase of mitosis or meiosis. This so-called chromosome delay may give rise to aneuploidy due to loss of chromosomes from the nucleus [7]. A number of studies have shown that DOX induces the formation of micronuclei both in vivo and in vitro in different cell lines [8–10]. Dhawan et al. [11] showed that DOX exerted both aneugenicity (generation of daughter cells with abnormal chromosome numbers) and clastogenicity (generation of daughter cells with chromosome/DNA breaks) in human lymphocytes. Additionally, the aneugenic and clastogenic potential of DOX was shown in Swiss Albino mice [12]. DOX clastogenicity has been reported in H9c2 cardiac cell line using the comet assay [13]. Furthermore, the CBMN assay is a multi-end-point assay for measuring DNA damage, including the induction of micronuclei via acentric chromosome fragments and whole chromosomes, which can be distinguished by kinetochore or centromere detection using molecular methods; cytotoxicity and apoptosis are also detected [14].

It is well documented that most cytotoxic anticancer agents induce apoptosis, increasing the possibility that defects in apoptotic programs contribute to treatment failure [15]. Apoptosis provides an important cell control mechanism and is characterized by distinct morphological characteristics [16]. It is an important phenomenon in cancer chemotherapy since various anticancer drugs exert their antitumor effect by inducing apoptosis [17]. Evidence suggests that DOX causes apoptotic cell death [18]. It has been also reported that DOX activates p53-DNA binding; it has been proposed that p53 could play an important function in anthracycline cytotoxicity [19].

The aim of the present study is, by a combination of different methods, to further clarify the genetic effect mechanism of DOX on different human cell types; i.e. primary, peripheral lymphocytes versus transformed cells, HL-60 leukemic cells. DNA damage was firstly conducted in HL-60 leukemic cells by alkaline comet assay, also modified with Fpg and hOGG1 repair enzymes. In parallel, a study was performed on micronucleation in both human cell systems using the CBMN cytome assay, in order to see possible differences on micronucleus induction due to the different characteristics of these cells (primary versus transformed). The mechanism of micronucleation was further studied by the FISH assay with pancentromeric probe in human lymphocytes in order to determine the micronucleus content. It is known that micronuclei containing acentric chromosome fragments originate due to chromosome breakage, while micronuclei containing whole chromosomes originate due to aneuploidogenic mechanisms. To clarify possible aneugenic effect of DOX, the organization of mitotic spindle was investigated by double immunofluorescence of the centrosome (γ-tubulin) and microtubules (β-tubulin). Apoptosis was studied on the HL-60 cells using the same multi endpoint assay, CBMN. The elucidation of the apoptosis mechanism in HL-60 cells was further investigated by Western blotting analysis of the caspase-3 levels, which plays a key role in the cascade of molecular events during programmed cell death. To the best of our knowledge, it is the first time that such a multifaceted investigation of this kind is conducted. From this point of view, our study will contribute to the knowledge on the genotoxic effects of DOX in human cells.

## Methods

**Fig. 1:**
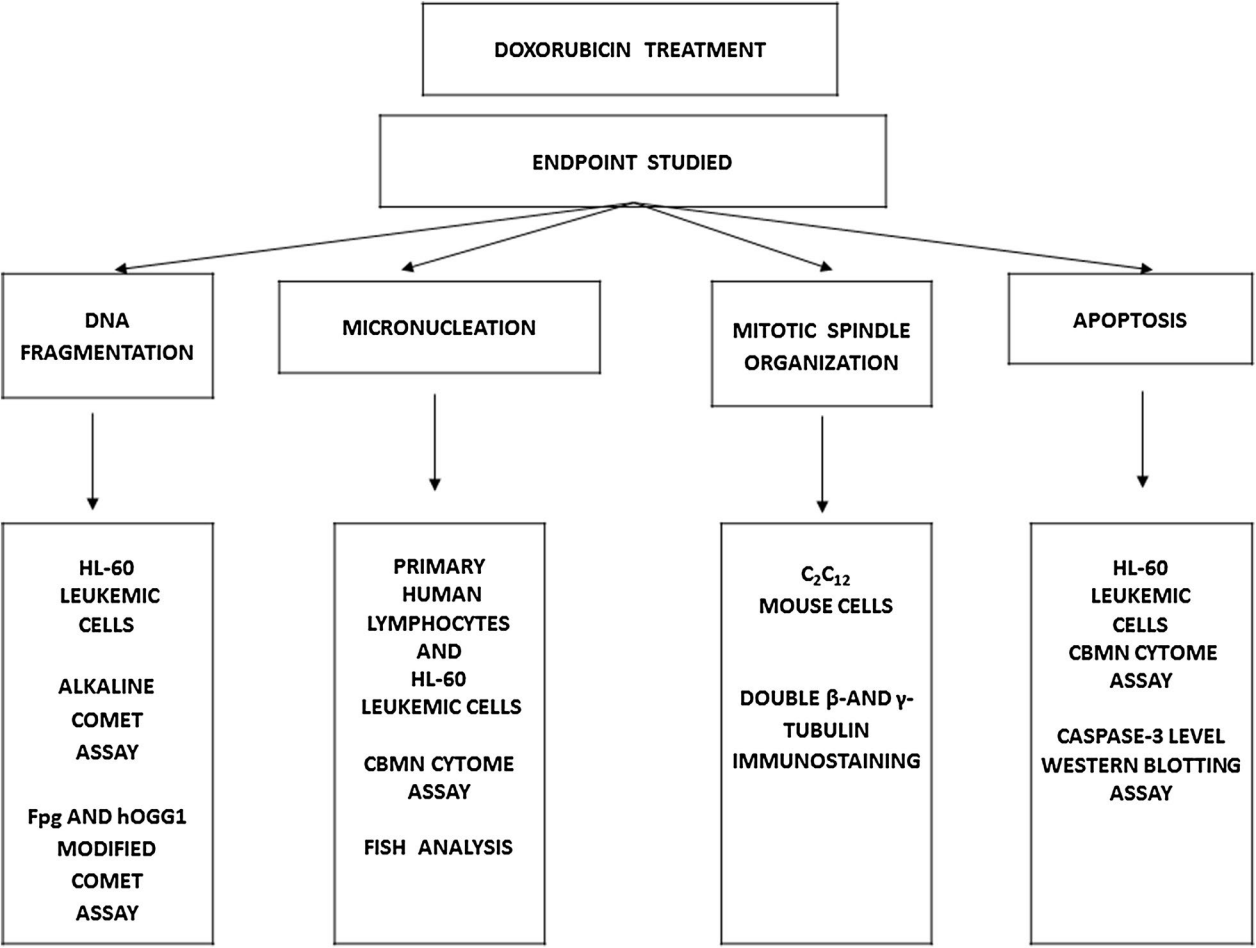
Genetic endpoints studied in different cell types and methodology followed

Figure 1 illustrates the experimental design of the study by presenting the analytical approaches performed in each cell type.

### Chemical compound

DOX was purchased from MP Biomedicals (LLC, CA, USA) [CAS. No. 25316-40-9] and was dissolved in double distilled water. The solvent concentration in cultures was always less than 0.5%.

### Cell cultures

#### HL-60 cell line

HL-60 is a human leukemic cell line (DSMZ, ACC3, Germany), derived from the peripheral blood leukocytes of an untreated 36 years old female with acute promyelocytic leukemia [20]. They are round single cells in suspension. They were routinely grown in RPMI 1640 medium (Biochrom AG, Berlin, Germany, F1215) supplemented with 10% fetal bovine serum (Biochrom AG, Berlin, Germany, S0113) and 1% penicillin–streptomycin (Biochrom AG, Berlin, Germany, 2213), at 37 °C in a 5% CO_2_ in humidified air. HL-60 leukemic cells were seeded at a cell density of 0.5 × 10^5^ cells ml^−1^ in 25 cm^2^ flasks.

#### Human lymphocyte cultures

After informed consent, two healthy males less than 30 years old, which were non-smoking, had received no treatment in the last 6 months, had not been exposed to X-rays and had no evidence or history of infection prior to the investigation, were used as blood donors to establish whole blood lymphocyte cultures.

### Study of DNA breakage

#### Alkaline comet assay

After 48-h culture initiation, HL-60 cells were treated with 0.5, 1.0, 2.0, 2.5 and 3.0 μg ml^−1^ DOX. Then, 6 h after treatment 25 µl of cells from 2 × 10^5^ cells ml^−1^, was suspended in 325 µl Low-Melting agarose (LM) 0.5% (Promega, Southampton, UK, V211A), and 75 μl of this mixture was immediately placed on a CometSlide (Trevigen, Gaithersburg, MD, USA), and the slides were placed flat in a refrigerator at 4 °C until the appearance of a clear ring at the edges of CometSlide sample area at the end of the gelling time. The slides were submerged gently in cold lysis buffer for 1 h at 4 °C (pH = 10), rinsed in distilled water, placed in a horizontal gel electrophoresis tank (Scie-Plas, UK) and covered with freshly prepared alkaline solution (pH > 13) for 30 min at 4 °C, to allow the DNA to unwind. Electrophoresis was carried out at constant 5 V cm^−1^ for 30 min at 4 °C (using a Consort, Belgium, EV261 power supply). The slides were rinsed twice with distilled water, washed in 70% ethanol for 5 min and then air-dried. Slides were further dried overnight with silica gel orange beads (Applichem, Darmstadt, Germany, A4569), and were stained with 2 µg ml^−1^ ethidium bromide and immediately photographed at 10 × 10 magnification. All steps were performed in the dark to avoid any light-induced DNA damage. As a positive control, hydrogen peroxide was applied at 100 µM for 20 min at 37 °C in a 5% CO_2_ in humidified air.

#### Fpg and hOGG1 modified comet assay

HL-60 cells were pretreated with DOX at 0.5 and 2.0 μg ml^−1^. The cell-agarose suspension slides were prepared as previously described for the standard comet assay. After lysis, the slides were washed three times, 5 min each time, with the enzyme buffer at room temperature. Fpg (Biolabs, Ipswich, MA, USA, MO240S) and hOGG1 (Biolabs, Ipswich, MA, USA, MO241S) excision repair enzymes were added to the gel (in 50 μl of enzyme buffer) at dilutions 1:1000 and 1:100, respectively. Also, control slides were treated with 50 μl of enzyme buffer alone. Gels were covered with a cover slip and incubated in humidified chamber/moist box for 45 min at 37 °C, and after incubation the procedure was followed as above.

#### Analysis of comets

Comet assay analysis was performed on at least 80 comets per slide with the CometScore 1.5 software (http://www.autocomet.com/products_cometscore.php). Comets at the edges of the slide were not analysed. Quantification of possible DNA damage was carried out using the parameter %DNA in tail as a measure of DNA migration in the tail of the comet.

### Micronucleation study

#### Human lymphocytes

MΝ analysis in human lymphocytes was performed with the CBMN assay, according to a published procedure [21]. Separate cultures were established corresponding to the different experimental points, control and treated. Whole blood (0.5 ml) was added into the culture medium consisting of 6.5 ml Ham F-10 (Biochrom, AG, Berlin, Germany, F0715), 1.5 ml fetal bovine serum (Biochrom, AG, Berlin, Germany, S0113), enriched with 1% penicillin–streptomycin (Biochrom, AG, Berlin, Germany, A2213) and 1% Glutamine (Biochrom, AG, Berlin, Germany, K0282). Cultures were grown at 37 °C in a 5% CO_2_ atmosphere with 95% humidity. Two cultures were established for each experimental point and for each donor. Lymphocytes were stimulated to divide with 60 μg ml^−1^ phytohemagglutinin (PHA, Biochrom, AG, Berlin, Germany, M5030) at the onset of the cultures. DOX was added into the cultures 41 h after culture initiation to give final concentrations 0.01, 0.025 and 0.05 μg ml^−1^. Cytochalasin-B (Sigma, St Louis, MO, USA, C6762), 6 μg ml^−1^, was added into the culture medium 44 h after culture initiation. Cells were harvested 72 h after culture initiation by centrifugation for 10 min. A mild hypotonic treatment with a solution of Ham F-10:ddH_2_O = 1:1 was given for 3 min at room temperature, and was followed by a 10-min fixation with a fresh solution of methanol:acetic acid = 3:1. Cell drops were layered onto clean slides added from a very low distance. Slides were stained with May-Grünwald (Merck, Darmstadt, Germany, 1-01424) and 10% Giemsa (Merck, Darmstadt, Germany, 09204), or were stored in the dark at 4 °C for FISH analysis. The Cytokinesis-Block Proliferation Index (CBPI) was determined by the equation CBPI = M_1_ + 2M_2_ + 3(M_3_ + M_4_)/N, where M_1_, M_2_, M_3_ and M_4_ correspond to the numbers of cells with one, two, three and four nuclei, respectively, and N the total number of analyzed cells. At least 2000 cells were evaluated for the identification of CBPI per each experimental point and per donor. To determine MN frequency at least 1000 binucleated cells were evaluated per each experimental point and per donor. Standard criteria were used for the identification of MN [7].

#### FISH analysis with pancentromeric probe in human lymphocytes

Human lymphocyte cultures were established from peripheral blood of the same donors and treated with DOX at 0.05 μg ml^−1^. FISH was performed using an α-satellite DNA probe for all human centromeres directly labeled with Fluorescein isothiocyanate (FITC) (Q-Biogene-MP, Biomedicals, France, PAHC0001-G) according to the company’s proposed protocol. Counterstaining was performed using 4′,6-diamino-2-phenylindole (DAPI) (Sigma, St Louis, MO, USA, CAS no. 47165-04-8) after which the slides were air-dried and mounted in Vectashield Mounting Medium (Vector, Burlingame, USA, CAS no. 152522-08-2). Vincristine sulfate (Sigma, St Louis, MO, USA, CAS no. 2068-78-2) at 0.03 μg ml^−1^ was used as a positive control. The slides were kept for long storage in the dark at 4 °C. Slides hybridized with the pancentromeric probe were analyzed for the presence or the absence of a centromere signal inside micronuclei for each experimental point. A centromere positive micronucleus (C^+^MN) was recorded when the signal had the same staining intensity as the main nucleus. MN were characterized only in those cells whose nuclei contained clear pancentromeric signals. A centromere negative micronucleus (C^−^MN) exerted no centromere signal. At least 40 MN were analyzed for the presence of a centromere-positive signal per donor and per experimental point.

#### HL-60 cells

Moreover, the CBMN assay was also used to assess the micronucleation on HL-60 cells due to DOX activity. As HL-60 cells have a variable doubling time from 20 to 45 h in an actively growing culture [22], DOX was added into the cultures 15 h after culture initiation at final concentration 0.5 and 2.0 μg ml^−1^, and cells were treated for 6 h. At the end of DOX exposure, HL-60 cells were washed once with Phosphate Buffered Saline (PBS) solution, and then suspended in freshly prepared complete medium containing 3.0 μg ml^−1^ cytochalasin-B. Subsequently, the cells were incubated for 28 h. The procedures for harvesting and fixing cells were followed as stated in the lymphocyte CBMN assay. At least 1000 binucleated cells were evaluated per each experimental point per culture to determine MN frequency. A minimum of 2000 cells were evaluated in order to determine the CBPI. Cytotoxicity of DOX was calculated by the cytotoxicity equation = 100 − 100 [CBPIT − 1/CBPIC − 1] where CBPIT and CBPIC is the CBPI of treated cells and untreated cells, respectively.

### Study of mitotic spindle organization—fluorescence double staining for β- and γ-tubulin

C_2_C_12_ cell line (DSMZ, ACC565) was derived from mouse myoblasts [23]. Cells were grown in a monolayer in essential medium and were routinely maintained in Dulbecco’s Modified Eagles Medium (DMEM) (Biochrom, AG, Berlin, Germany, F0415) supplemented with 20% fetal bovine serum (FBS) (Biochrom AG, AG, Berlin, Germany, S0115), gentamycin (Gibco-Invitrogen, Grand Island, NY, CAS no. 1403-66-3) and fungizone (Gibco-Invitrogen, Grand Island, NY, CAS no. 1397-89-3), at 37 °C in a 5% CO_2_ in humidified air.

To study the effect of DOX on mitotic spindle organization, the cells were grown at a density of 1.8 × 10^5^ cells ml^−1^ on 22 × 22 mm glass coverslips in 35 mm Petri dishes and treated with DOX at 0.05 μg ml^−1^ for 48 h. Demecolcine (Sigma, St Louis, MO, USA, CAS no 477-30-5) at 0.005 μg ml^−1^ was used as a positive control. The study of mitotic spindle morphology was performed by fluorescence double staining for *β*- and *γ*-tubulin. Cells were washed once in cold PBS and then incubated overnight at 4 °C with a 1:200 diluted mouse anti-γ-tubulin monoclonal antibody (Sigma, St Louis, MO, USA, T6557). Following rinsing with PBS, slides were incubated for 50 min in a humidified chamber at 37 °C with a 1:20 diluted FITC-conjugated secondary anti-mouse IgG fragment antibody from sheep (Sigma, St Louis, MO, USA, F2266). After extensive washing in cold PBS, cells were kept for 50 min in the presence of the blocking reagent Goat Serum (Sigma, St Louis, MO, USA, S9023). Cells were washed twice in cold PBS and incubated overnight at 4 °C with a 1:100 diluted mouse anti-*β*-tubulin monoclonal antibody (Sigma, St Louis, MO, USA, T4026). Finally, samples were incubated for 50 min at 37 °C with an anti-mouse antibody conjugated with tetramethylrhodamine (TRITC) from goat 1:20 diluted (Sigma, St Louis, MO, USA, T7782). DNA was counterstained with DAPI (Sigma, St Louis, MO, USA, CAS no. 28718-90-3). One thousand cells were analyzed to estimate Mitotic Index as percentage (%) of cells in mitosis. The ability of DOX to arrest cells in metaphase was evaluated by the distribution of mitotic figures in at least 650 mitotic cells. A total of at least 159 and maximum 474 metaphases were analyzed for the presence of *β*- and *γ*-tubulin reflecting chromosome orientation as well as the number of centrosomes, respectively.

### Apoptosis study

#### Apoptotic cell frequency

Analysis of apoptotic HL-60 cells was performed with the CBMN assay. Cell cultures were established as in micronucleation study. Cells were treated with DOX at two different concentrations, 0.5 and 2.0 μg ml^−1^ for 6 h. Slides were stained with May-Grünwald and 10% Giemsa. Standard criteria were used for the identification of apoptotic cells [14].

Complementary to the above, caspase-3-level-Western blotting analysis was achieved. HL-60 cells were treated with DOX at the same concentrations and time as above. The procedure was followed according to recent publication [24]. Caspase-3 and β-actin were detected with primary antibodies for caspase-3 (1:1000, Cell Signaling, Boston, MA, USA, 9665) and β-actin (1:1000, Cell Signaling, Boston, MA, USA, 4967). The protein amount in the bands of each lane was quantified by densitometric analysis relative to the actin numerical quantities. In each independent experiment, protein amount variation fold was calculated considering equal to 1 the protein/β-actin ratio observed in untreated cells [25, 26].

### Microscopic analysis

Microscopic analysis of FISH signals, immunofluorescence detection for *β*- and *γ*-tubulin and comets was performed on a Zeiss axioscop epifluorescence microscope equipped with a high-sensitive monochrome CCD-camera as well as with an image analysis system (ISIS MetaSystem, Newton, MA, USA). The band pass filters used were 546, 490 and 360 nm for green, blue and ultraviolet light, respectively.

### Statistics

Statistical analysis of data from the comet assay was performed using one-way ANOVA (OriginPro, version 8.5, Stoke Mandeville, UK). *p* value at < 0.05 was considered to be statistically significant. Statistical analysis of data in MN, apoptosis and mitotic spindle study was achieved by the G-test for independence on 2 × 2 tables. This test is based on the general assumption of the χ^2^ analysis, but offers theoretical and computational advantages.

## Results

### Induction of DNA strand breaks in HL-60 cells

**Fig. 2:**
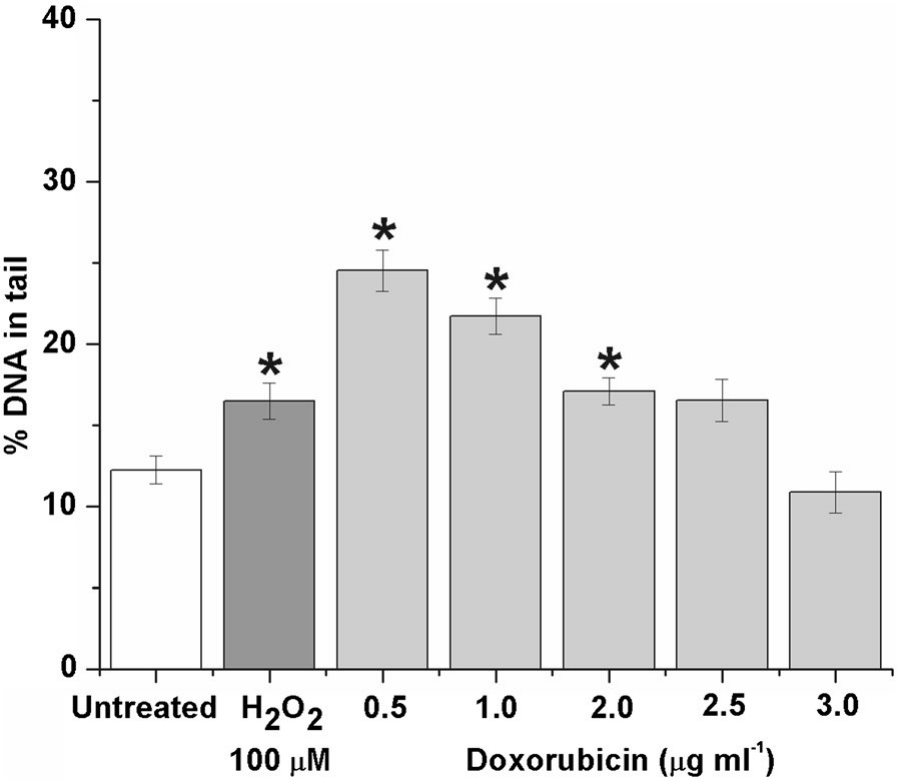
% DNA in tail in HL-60 comets after treatment with various concentrations of doxorubicin. H_2_O_2_ (100 μM) was used as positive control. DNA was stained with ethidium bromide. *p ≤ 0.05 in comparison with control (one-way ANOVA). Bars represent standard error

Treatment of HL-60 cells with DOX resulted in a statistically significant increase in the studied parameter (% DNA in tail) indicating the generation of DNA damage. Indeed, % DNA in tail was statistically higher at 0.5, 1.0 and 2.0 than in control (Fig. 2) but not at 2.5 and 3.0 μg ml^−1^. Treatment of cells with 100 μΜ Η_2_Ο_2_, which was used as positive control, resulted in a statistical increase for the studied parameter.

**Fig. 3:**
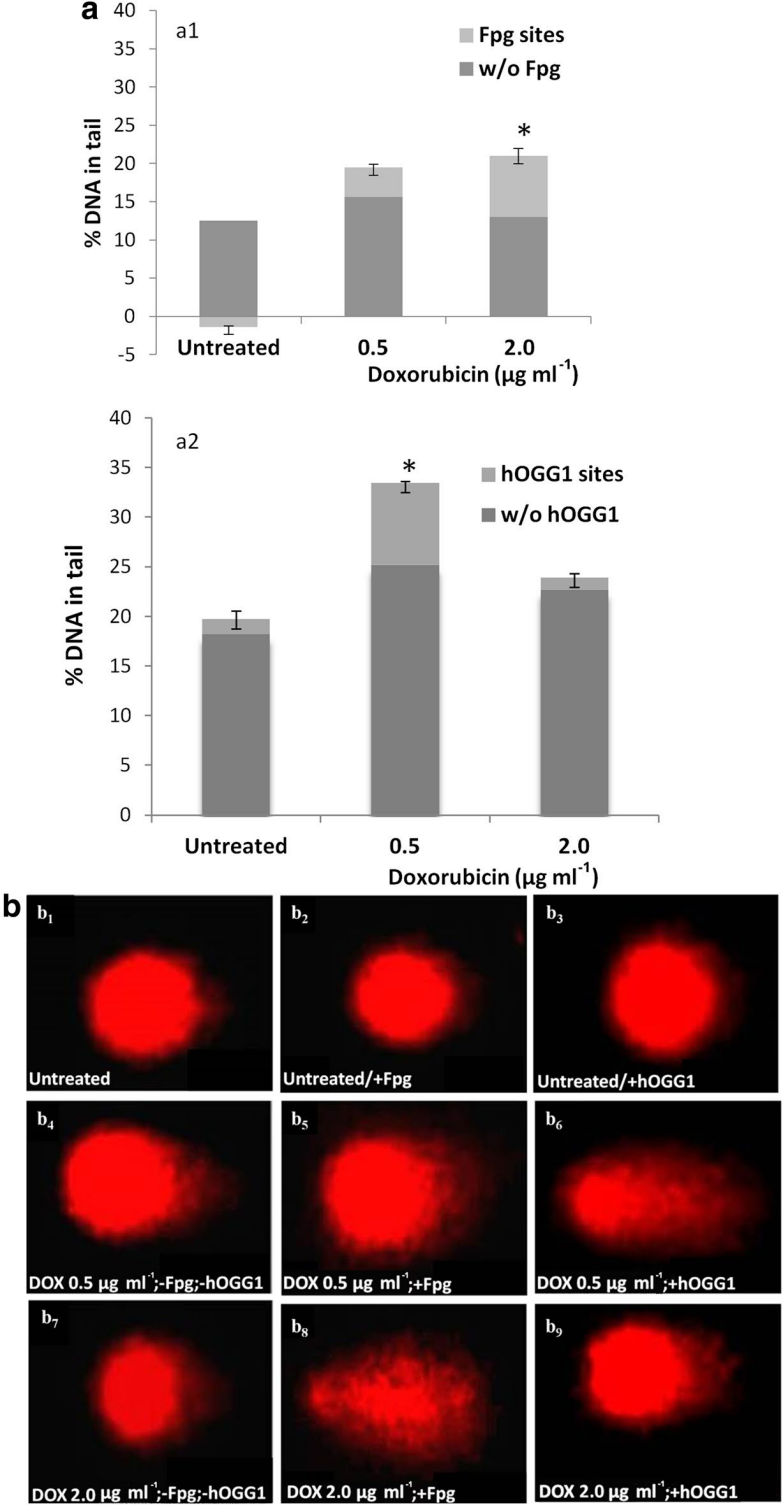
% DNA in tail in HL-60 comets after treatment with doxorubicin and incubation of nucleoids with Fpg or hOGG1 endonuclease. Data represent values generated by Fpg (**a1**) and hOGG1 (**a2**) incubation, that were calculated by subtracting the value of DNA damage caused in the HL-60 cells that were not treated with the respective enzyme from the DNA damage caused in cells incubated with the enzymes. Subtracted values are pointed out in light grey area marked ‘‘Fpg sites’’ and “hOGG1 sites”. **b** HL-60 comets, no doxorubicin treatment/no endonuclease incubation (**b**_**1**_); no doxorubicin treatment/Fpg endonuclease incubation (**b**_**2**_); no doxorubicin treatment/hOGG1 endonuclease incubation (**b**_**3**_); doxorubicin treatment at 0.5 μg ml^−1^/no endonuclease incubation (**b**_**4**_); doxorubicin treatment at 0.5 μg ml^−1^/Fpg endonuclease incubation (**b**_**5**_); doxorubicin treatment at 0.5 μg ml^−1^/hOGG1 endonuclease incubation (**b**_**6**_); doxorubicin treatment at 2.0 μg ml^−1^/no endonuclease incubation (**b**_**7**_); doxorubicin treatment at 2.0 μg ml^−1^/Fpg endonuclease incubation (**b**_**8**_) and doxorubicin treatment at 2.0 μg ml^−1^ /hOGG1 endonuclease incubation (**b**_**9**_). DNA was stained with ethidium bromide. * *p *≤ 0.05 in comparison with untreated cells (one-way ANOVA). Bars represent standard error

To further analyze the aforementioned results, we employed the modified comet assay using the Fpg and hOGG1 repair enzymes. This assay is based on the following: digestion of the nucleoid DNA after lysis with excision repair enzymes (Fpg or hOGG1) converts small alterations to DNA bases to strand breaks. In particular, both Fpg and hOGG1 act both as an *N*-glycosylase and an AP-lyase. The *N*-glycosylase activity releases damaged purines/pyrimidines from double stranded DNA, generating an AP site and the AP-lyase activity cleaves to the modified base thereby removing the AP site and leaving a 1 base gap. The repair mechanism does not continue, and DNA is drawn towards to anode, forming a comet tail during electrophoresis. The Fpg enzyme cuts both at 3′ and 5′ ends, while the hOGG1 enzyme cuts only at the 3′ end to the modified base [27–29]. HL-60 cells were exposed to the lowest and highest DOX concentration, 0.5 and 2.0 μg ml^−1^, respectively, that induce DNA breakage in the standard comet assay. A significant increase of strand breaks was observed, following treatment with the endonuclease Fpg at 2.0 μg ml^−1^ DOX, since % DNA in Tail was statistically significantly increased in comparison with cells not treated with DOX (Fig. 3a_1_). On the other hand, incubation of HL-60 cells with the hOGG1 endonuclease led to a statistically significant increase of strand breaks at 0.5 μg ml^−1^ DOX in comparison with the cells that were not treated with DOX (Fig. 3a_2_). Fluorescence images of HL-60 comets are shown in Fig. 3b.

### Induction of micronucleation

#### Human lymphocyte cultures. Micronucleus frequency-FISH analysis

**Fig. 4:**
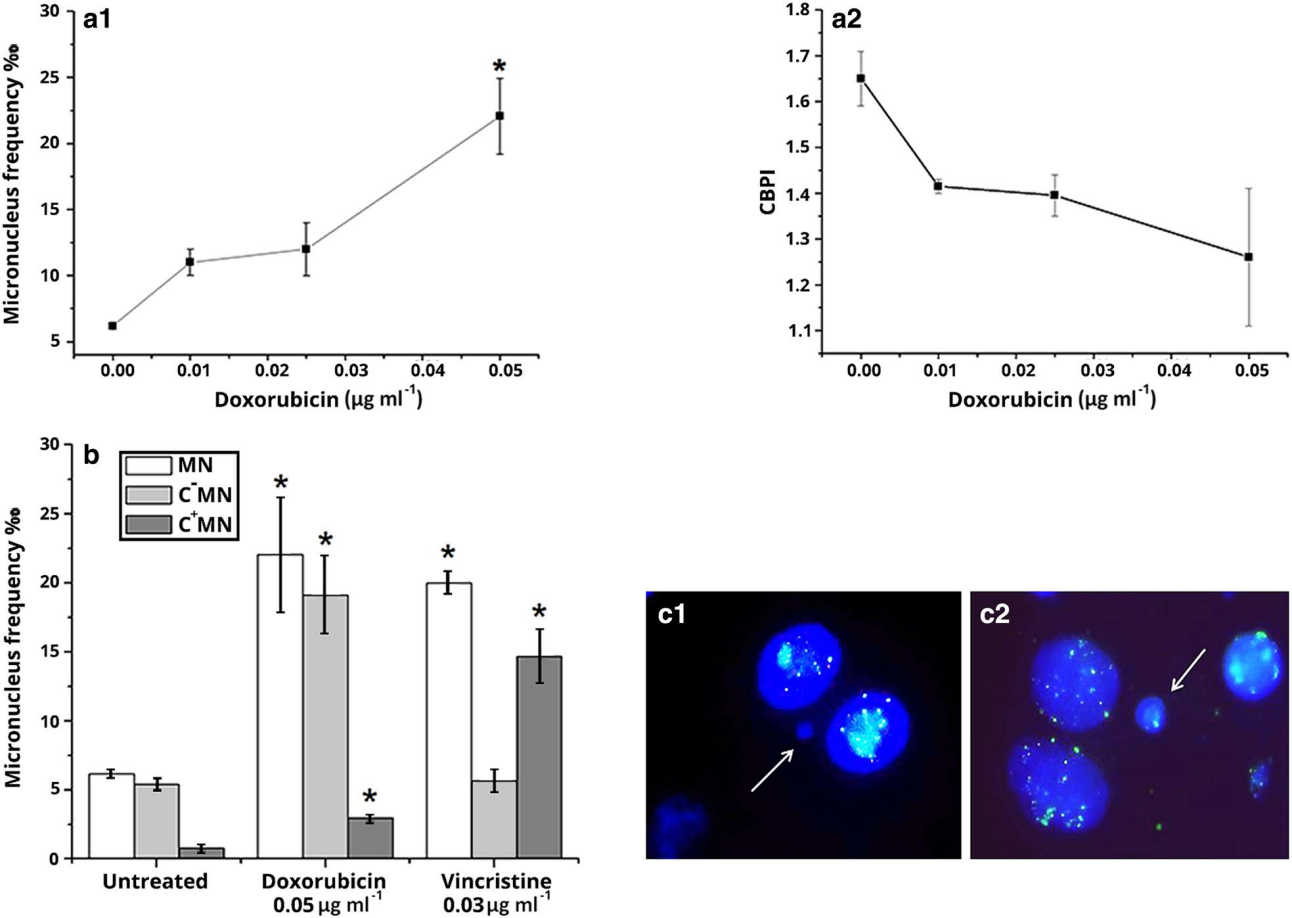
**a**_**1**_ Micronucleus frequency in human lymphocytes treated with doxorubicin. **a**_**2**_ Cytokinesis Block Micronucleus Index (CBPI) in human lymphocytes treated with doxorubicin. **b** FISH analysis with pancentromeric probe. **c** Lymphocytes with C^−^MN (**c**_**1**_) and C^+^MN (**c**_**2**_). (C^−^MN: micronucleus without centromeric signal, C^+^MN: micronucleus exerting centromeric signal. * *p *≤ 0.05 in comparison with control (G-test). Vincristine sulfate was used as a positive control. The values represent the average of the two donors. Bars represent standard error

Preliminary experiments that were performed on peripheral human lymphocytes showed that treatment of lymphocytes with DOX at concentrations higher than 0.075 μg ml^−1^ resulted to a high cytotoxicity (above 80%). Thus, we did our experiments with human lymphocytes at lower concentrations, than those studied on HL-60 leukemic cells, 0.01, 0.025 and 0.05 μg ml^−1^, at which cytotoxicity was at significantly lower levels. As it is shown in Fig. 4a1, the frequency of human lymphocytes containing MN was increased statistically significantly at 0.05 μg ml^−1^ DOX. A reduction in CBPI was also observed (Fig. 4a2). FISH analysis of binucleated cells was performed at 0.05 μg ml^−1^, at which the higher MN frequency was determined. Pancentromeric probe was used to detect the presence or absence of centromere inside the micronuclei. The absence (C^−^MN) or the presence (C^+^MN) of a centromere signal in micronuclei, correspond to the inclusion of chromosome fragment or whole chromosome, respectively. Micronuclei that did not exert FISH signal (C^−^MN) was the main type of induced MN observed. In addition, the frequency of micronuclei exerting FISH signal (C^+^MN) was increased (Fig. 4b), although at a lesser level. Additionally, treatment of cells with the well-known aneugen vincristine (at 0.03 μg ml^−1^) resulted in high frequency of C^+^MN cells. Human lymphocytes with MN are shown in Fig. 4c.

#### HL-60 cells. Micronucleus frequency and apoptosis

**Fig. 5:**
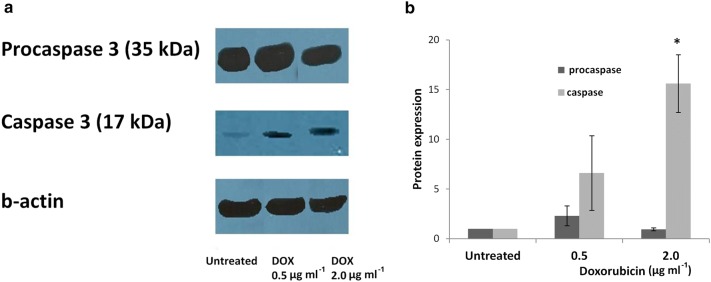
**a** Western blots and **b** densitometric quantification, showing the caspase-3 level in HL-60 cells after treatment with doxorubicin. * *p *≤ 0.05 in comparison with control (G-test). Bars represent standard error

The analysis of the comet assay parameter,  % DNA in tail, revealed that DOX exerts clastogenic activity at 0.5, 1.0 and 2.0 μg ml^−1^. Therefore, we further investigated the ability of DOX to induce micronucleation and apoptosis, both at lowest and highest concentrations (0.5 μg ml^−1^ and 2.0 μg ml^−1^, respectively) at which DNA breakage was induced by DOX. A statistically significant induction of apoptosis was observed after treatment of HL-60 cells with 0.5 and 2.0 μg ml^−1^ DOX. This induction is more potent at the higher concentration of 2.0 μg ml^−1^. On the other hand, DOX induced statistically significant increase of micronucleus frequency at 0.5 μg ml^−1^, while at 2.0 μg ml^−1^ it did not provoke micronucleation (Table 1). Furthermore, possible alteration of the caspase-3 level was followed by Western-blot analysis in HL-60 cells exposed to DOX. The primary antibody for caspase-3 detects endogenous levels of full-length protein (procaspase-3, 35 kDa), as well as the large fragment (17/19 kDa) of caspase-3 resulting from cleavage at aspartic acid 175. An elevated level of caspase-3 was observed when HL-60 cells were exposed to DOX at 0.5 and 2.0 μg ml^−1^. This elevation is more pronounced at the highest concentration (2.0 μg ml^−1^) with parallel decrease of procaspase-3 level (Fig. 5a, b).

**Table 1:** Induction of apoptosis and micronucleation in HL-60 cells after treatment with doxorubicin

Doxorubicin (μg ml^−1^)	Induction of apoptosis			Total cells	Micronucleus Induction		
	Total cells	Live cells (%) Mean ± SE	Apoptotic cells (%) Mean ± SE		Cells with MN (%) Mean ± SE	CBPI	Cytotoxicity (%) Mean ± SE
0	9262	8507 92.43 ± 2.55	748 7.5 ± 2.51	5122	55 1.1 ± 0.19	1.55 ± 0.07	–
0.5	10,614	4260 39.83 ± 4.48	6220 58.88* ± 4.35	2329	47 3.86* ± 1.87	1.22 ± 0.03	60.01 ± 3.35
2	10,673	1417 12.95 ± 2.81	8587 80.51* ± 0.56	483	8 1.3 ± 0.39	1.28 ± 0.07	50.61 ± 9.77

*MN* micronucleus, *CPBI* Cytokinesis Block Proliferation Index, *SE* standard error

* *p *≤ 0.0001 in comparison with control (G-test)

### Mitotic spindle analysis

#### Mitotic progression and abnormal metaphases

**Fig. 6:**
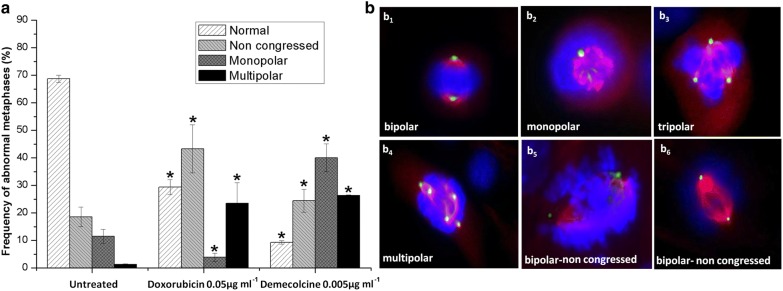
**a** Frequency of abnormal C_2_C_12_ metaphases in relation to γ-tubulin signals and chromosome orientation. **b** Distinct types of metaphase cells: normal metaphase (**b**_**1**_); monopolar metaphase (**b**_**2**_); tripolar metaphase (**b**_**3**_); multipolar metaphase (**b**_**4**_); and bipolar non-congressed metaphases (**b**_**5–6**_). * *p *≤ 0.05 in comparison with control (G-test). Demecolcine was used as positive control. Bars represent standard error

This analysis cannot be performed on human lymphocytes and HL-60 cells or any other cell system cultured in suspension, because it should be done in cells that are divided by adhering to a solid substrate [30]. Therefore, we used the C_2_C_12_ cell line for such analysis, since these cells divide by adhering to the plastic substrate of the flask, and they have the ability to form an extensive microtubule network. Thus, they constitute an appropriate cell system to study alteration in the organization of mitotic apparatus, and this system was chosen for this purpose. In Table 2, it is shown that DOX is not able to provoke metaphase arrest, since no statistically significant increase of the percentage of metaphase cells was observed. However, treatment of cells with 0.05 μg ml^−1^ DOX led to a statistically significant increase of ana-telophases in comparison with the untreated cultures, indicating an accumulation of the cells at this mitotic stage. The estimation of Mitotic Index revealed that DOX caused delay of cell cycle proliferation, since a statistically significant decrease of Mitotic Index was observed in comparison to the control. Total abnormal mitotic cells calculated as percentage of mitotic cells with abnormalities (reflecting the number of γ-tubulin signals and chromosome orientation on the number of total mitotic cells scored) were increased after DOX treatment. The same holds true for the total abnormal metaphases, expressed as percentage of metaphases with abnormalities on total metaphases scored. Additionally, metaphase cells were independently classified to abnormal figures, as shown in Fig. 6a. After exposure of cells to 0.05 μg ml^−1^ DOX, normal metaphases were significantly decreased, and a statistically significant increase of bipolar non-congressed and multipolar metaphases was observed in comparison to the control cultures. All types of abnormal metaphases are also induced by the aneuploidogenic compound demecolcine, which was used as positive control. Abnormal C_2_C_12_ metaphases are shown in Fig. 6b.

**Table 2:** Mitotic figure distribution and the effect on the M.I. in C_2_C_12_ cells treated with doxorubicin

Chemical compound (μg ml^−1^)	Total mitotic cells	Prophases (%) Mean ± SE	Metaphases (%) Mean ± SE	Ana-telophases (%) Mean ± SE	Total abnormal mitotic cells (%) Mean ± SE	Total abnormal metaphases (%) Mean ± SE	Mitotic Index (%) Mean ± SE
0	1436	24 1.69 ± 0.25	474 32.88 ± 2.03	938 65.44 ± 1.78	152 10.51 ± 1.12	149 10.51 ± 1.07	5.86 5.87 ± 0.57
DOX 0.05	658	5 0.79^*^ ± 0.79	159 24.15^*^ ± 0.49	494 75.07^*^ ± 0.31	477 72.69^*^ ± 5.54	112 17.04^*^ ± 0.32	1.34 1.34^*^ ± 0.03
DEM 0.005	650	9 1.38^*^ ± 0.06	425 64.85^*^ ± 7.57	216 33.77^*^ ± 7.62	409 62.49^*^ ± 6.20	385 58.78^*^ ± 6.46	2.14 2.14^*^ ± 0.07

*DOX* doxorubicin, *DEM* demecolcine was used as positive control, *M.I.* Mitotic Index, *SE* standard error

* *p *≤ 0.0001 in comparison with control (G-test)

## Discussion

The ability of DOX to induce DNA damage was analysed in HL-60 cells using alkaline comet assay. DOX provoked DNA migration from the nucleus. The generation of damage on DNA strands seems to be more potent at low DOX concentrations. The ability of DOX to induce fragmentation of genetic material is correlated with the oxidation of DNA bases resulting in the formation of alkali labile sites. Accordingly, DNA damage on various cell types using comet assay has been previously reported [31]. Incubation of lysed cells with endonucleases specific for certain lesions, e.g. hOGG1 and Fpg, improved the specificity of the comet assay, since endonucleases recognize abnormal bases and generate additional breaks [6]. Excising the specific modified bases from DNA by these enzymes results in the formation of apurinic or apyrimidinic sites, which then are cleaved due to the ability of these endonucleases to act as AP-lyases, too. The generated gaps in the DNA strands, due to oxidation to DNA bases, can be then recognized by the comet assay. Indeed, our results showed increased DNA damage in HL-60 cells treated with DOX, when lysed cells were incubated with hOGG1 and Fpg endonucleases. Specifically, increased values of % DNA in tail were detected at 0.5 and 2.0 μg ml^−1^ DOX treatment after hOGG1 and Fpg post incubation, respectively. A possible explanation on the mechanism that leads to this result would be the generation of different kinds of oxidized bases, which are specifically recognized by hOGG1 at the lowest concentration and by Fpg at the highest concentration; hOGG1 recognizes almost exclusively 8-oxoG, while Fpg recognizes purine DNA oxidation and not only 8-oxoG [27, 32]. Several types of oxidized bases after cell exposure to DOX have been determined [33]. Exposure of MCF-10A breast epithelial cells to DOX resulted in a time-dependent formation of eleven oxidized DNA bases [34]. On the other hand, it has been reported that the increased incidence of DOX-induced DNA damage can be modulated by the complimentary use of the natural chemical squalene [35]. We also found that DOX is able to cause increased micronucleus frequency in human lymphocytes as well as in HL-60 cells. As the micronucleation is the result either of chromosome breakage or chromosome delay we considered interesting to proceed with FISH analysis in human lymphocytes to investigate the mechanism of micronucleus generation rather than repeat comet assay. DOX exerted both chromosome breakage and, at a lesser extent, chromosome delay at least at the studied concentration (0.05 μg ml^−1^). This was observed by the increased frequencies of centromere-negative and centromere-positive MN frequency in human lymphocyte cultures. It has been reported that DOX increased micronucleus frequency in cultured human lymphocytes in a dose-dependent manner, micronuclei being both kinetochore negative and positive, indicating clastogenic and aneugenic properties of DOX [11]. A dose-dependent increase of micronucleus frequency was also reported in the same cell system [36]. Additionally, DOX aneugenic effect was also observed in lymphocytes from healthy individuals and cancer patients, since an increase in the trisomy of chromosomes 7 and 17, using FISH DNA-specific probes was identified [37]. To better clarify the aneugenic effect of DOX in complement to the micronucleus analysis, we investigated the integrity of the mitotic apparatus. It was shown that DOX provoked high abnormal metaphases, that is, non-congressed and metaphases with abnormal centrosome numbers, possibly due to abnormal duplication or fragmentation of this organelle. These phenomena are related with abnormal chromosome segregation. It has been reported that in the majority of DOX-treated giant HCT116 cells centrosomes were visible at a single pole only and were often over-duplicated. Also, in the less frequent cells with centrosomes separated to two poles, their numbers were abnormally high, and clusters of centrosomes were visible rather than a single one per pole [38]. It is known that dysfunction of the centrosome results in spindle abnormality, leading to aberrant chromosome segregation and genetic instability [39]. However, there is evidence that DOX induced aneuploidy is significantly reduced by dexrazoxane that is administered to mitigate DOX-caused cardiotoxicity [40].

Finally, increased frequency of apoptosis was detected in HL-60 cells. Additionally, caspase-3 was activated in accordance with the morphological alterations in nuclei of HL-60 cells, which indicates the involvement of caspase-3 in the generation of apoptosis by DOX. This is supported by previous studies which have proposed that DOX is an inducer of apoptotic cell death [41]. Furthermore, the reconstitution of caspase-3 in MCF-7 cells resulted to sensitization of cells to DOX-induced apoptosis [42], while DOX enhanced caspase-3 level in a dose-dependent manner in HaCat cells [43]. It has been recently reported that siRNA inactivation of *TP53* and *RB1* genes in MCF-7 cells led to alteration on cell cycle phase distribution [44], while siRNA targeted *HdmX* or *Hdm2* genes sensitized MCF-7 cells to DNA damage [45]. On the other hand, Eom et al. [46] reported that different doses of DOX activate different regulatory mechanisms in inducing either apoptosis or cell death through mitotic catastrophe.

## Conclusions

In conclusion, the results of our study can be summarized as follows:

Comet assay analysis revealed DNA breakage by DOX, which was further increased after incubation of nucleoids with the Fpg and hOGG1 excision repair enzymes, indicating that DOX generates DNA lesions, due to DNA base oxidation, that are repaired by these enzymes.

DOX also provokes increase of MN frequency in human lymphocytes and HL-60 leukemic cells. Micronuclei are generated mainly through DNA breakage and at a lesser extent through chromosome delay, as was shown after FISH analysis in human lymphocytes. Analysis of mitotic spindle showed disturbance of chromosome orientation as well as centrosome duplication and/or separation, indicating abnormal chromosome segregation due to DOX. Increased frequency of apoptotic HL-60 cells was observed after treatment with various doses of DOX. Caspase-3 seems to be involved in the generation of apoptosis in HL-60 cells.

Considering all the above, our findings, derived from different treatment schedules, doses and time of exposure on different cell types (i.e. primary versus transformed cells) contribute to the better understanding of the mechanisms by which DOX induces genotoxic effects on human cells.

## Abbreviations

CBMN: Cytokinesis-Block Micronucleus assay
CBPI: Cytokinesis-Block Proliferation Index
DOX: doxorubicin
DEM: demecolcine
FISH: Fluorescence In Situ Hybridization
FITC: Fluorescein isothiocyanate
Fpg: formamidopyrimidine
hOGG1: human 8-oxoguanine
MN: micronucleus
PBS: Phosphate Buffered Saline
SCGE: Single Cell Gel Electrophoresis
TRITC: tetramethylrhodamine
